# Tobacco Stalk Flour/Magnesium Oxysulfate Whiskers Reinforced Hybrid Composites of Recycled Polypropylene: Mechanical and Thermal and Antibacterial Properties

**DOI:** 10.3390/polym14040815

**Published:** 2022-02-20

**Authors:** Qinghua Yuan, Wei Yang, Zhuwen Ma, Zhenrui Huang, Lin Cao, Zhidan Lin, Peng Zhang

**Affiliations:** 1 Key Laboratory of Crop Genetic Improvement of Guangdong Province, Guangdong Provincial Engineering & Technology Research Center for Tobacco Breeding and Comprehensive Utilization, Crops Research Institute, Guangdong Academy of Agricultural Sciences, Guangzhou 510640, China; qinghuay@foxmail.com (Q.Y.); mazhuwen@gdaas.cn (Z.M.); huangzhenrui@gdaas.cn (Z.H.); 2 Institute of Advanced Wear & Corrosion Resistant and Functional Materials, Jinan University, Guangzhou 510632, China; yangwei@stu2019.jnu.edu.cn (W.Y.); lincao@stu2018.jnu.edu.cn (L.C.)

**Keywords:** tobacco stalk, recycle polypropylene, magnesium oxysulfate whiskers, composites

## Abstract

The present investigation utilizes tobacco stalks flour and magnesium oxysulfate whiskers as fillers to enhancers the recycle polypropylene through melt blending and injection molding. Studied the microscopic morphology, mechanical, thermal, and antibacterial properties of recycled polypropylene (rPP) based composites with different weight ratios of tobacco stalks flour (TSF) and magnesium oxysulfate whiskers (MOSw). Composites’ morphological studies indicated that tobacco stalks flour, and recycled polypropylene has good adhesion, improving composites’ mechanical properties. The addition of TSF did not significantly change the tensile strength of rPP, but it can effectively increase the flexural strength and flexural modulus. Compared with rPP, adding 30 wt% tobacco stalks flour to rPP can increase the flexural strength by about 32.74%. Meanwhile, the addition of magnesium oxysulfate whiskers further improves the material’s tensile strength. An increase in tobacco stalks flour content in the rPP enhances the crystallization temperature and degree of crystallinity of the polymer. In addition, attributed to the existence of tobacco stalks flour hydrophilic and antibacterial ability, the water absorption of the hybrid composites was increased and obtained antibacterial ability. Hence, this study provides a new development idea for tobacco stalks r recycling and applications.

## 1. Introduction

The use of plastic products brings convenience to people’s daily lives but produces environmental pollution simultaneously [1]. Hence, plastics recycling has become a research hotspot in recent years [2]. The use of recycled plastics increases plastics’ added value, endow them with sustainability, and reduce environmental pollution caused by plastics [3]. As the second most consumed thermoplastic polymer globally, polypropylene is widely used in electrical appliances, automobiles, construction, medical, packaging, and fiber [4]. However, the discard rate, the second-highest globally, poses a serious threat to the environment. Notably, PP can be recycled and reused because of its excellent performance, low price, and unchanged structure and mechanical properties after multiple processing [5,6]. The recycling of PP can produce new value-added products with lower costs and reduce environmental pollution.

Due to the influence of light, heat, oxygen, or stress during use and the combined action of mechanical and heat such as grinding and shearing during the polymer recycling process, the polymer molecular chain segment will be broken oxidatively degraded [7,8]. In addition, degradation will reduce the appearance and performance of the recycled polymer, reducing its reusability. Therefore, to enhance the mechanical properties of recycled plastics, researchers often introduce mineral fillers, natural plant fibers, elastomers, and other fillers into the recycled polymer matrix [9]. Natural plant fibers have a wide range of resources, simple access, renewable, low cost, high strength, and other characteristics that have received extensive attention from researchers. Hence, selecting cheaper and easily available plant fiber sources as polymer enhancers has become a research hotspot [10].

Recently, among the numerous nature fillers, the wood fibers, non-wood fibers, semi-wood fibers, leaves, and stalks of crops were used as the filler of plant fiber /polymer composites. The sources are processing residues, agricultural waste, and crop straws. Such as wheat straw [11,12,13,14], rice husk [15,16,17], date palm powder [18,19,20,21], and mango wood waste [22] have been used as fillers and enhancers to improve the mechanical properties of PP. Meanwhile, there are also reports that ramie and Sabai grass fibers have provided antibacterial properties for polymer composites [23].

Tobacco stalks, as agricultural wastes, have high yields, accessible collection, low cost, and their cellulose content can reach 23% [24]. In addition, its extract has specific antibacterial properties [25,26]. However, the directly composted and returned to the field of tobacco stalks may cause the outbreak of tobacco diseases in the next year. Hence incineration becomes the typical treatment method of tobacco stalks. However, incineration may cause the waste of resources and environmental pollution [27]. Therefore, tobacco stalks could be added as a filler to recycled polypropylene. It could enhance the mechanical properties of polypropylene and bring antibacterial properties to composite materials while reducing resource waste and environmental pollution, providing ideas for new application prospects for tobacco stalks.

Although plant fiber as a polymer filler can effectively reduce the cost of materials, it is difficult to use as a load-bearing material because of the lower flexural and tensile properties than plywood and oriented strand board in load-bearing structural applications [28]. There are two significant ways to improve the mechanical properties of plant fiber/polymer composites. Firstly, coupling agents such as maleic anhydride grafted PP and silane coupling agents were used to enhance the interface compatibility of natural fibers and PP [29,30,31,32]. Second, some inorganic materials (glass fiber [18], carbon fiber [33], carbon nanotube [34], clay [35,36]) were filled to reinforce the mechanical properties of the polymer. However, few reports focus on co-filling reinforced plant fiber /polymer composites with natural cellulose fibers and needle-like mineral whiskers. The basic magnesium sulfate whiskers (MOSw), the main components are Mg(OH)_2_ and MgSO4, are particularly suitable for the enhancer of plastics. Meanwhile, MOSw is inexpensive with flame retardancy properties [37,38,39]. It’s an ideal filling material to reinforce the mechanical properties of plant fiber /polymer composites.

To handle the problem of difficulty of recycling tobacco stalks, the environmental pollution by tobacco stalks incineration, and the low flexural and tensile properties of natural fiber/polymer composites. In this manuscript, we used recycled polypropylene (rPP) as the base material, tobacco stalks flour (TSF), and basic magnesium sulfate whiskers (MOSw) as fillers. The mechanical, thermal, water absorption properties of TSF/rPP composites with different mass fractions of TSF have been studied. Meanwhile, the basic magnesium sulfate whiskers were used as enhancers to improve further the tensile and flexural properties of TSF/rPP.

## 2. Materials and Methods

### 2.1. Materials

Recycled PP was supplied by Run Feng Sci.&Tech in the form of pellets. The tobacco stems flour was provided by the Crops Research Institute Guangdong Academy of Agricultural Sciences. Tobacco stalks are peeled and washed after removed the inner core. Then, tobacco steams pieces were dried in a blast drying box at 70 °C for 24 h, after that they were ground to 50 mesh. Basic magnesium sulfated whisker, with 2 μm diameter and aspect ratio of 8–70, offered from the Jiangxi Fengzhu New Materials Technology CO., LTD. Maleic anhydride grafted PP (MAH-g-PP, containing 1.2 wt% MA) was supplied by Kangjin New Material Technology Co., Ltd (Dongguan, China).

### 2.2. Composite Preparation

The formulations of the filled thermoplastic composites are presented in Table 1. In order to improve the compatibility of fillers with rPP, grafting maleic anhydride MAH onto PP (PP-g-MAH) was used as a compatibilizer to be added to the composites. All materials were prepared by melt compounding. TSF, rPP, MAH-g-PP, and MOSw were dried at 70 °C for 2 h in a blast drying box before the extrusion. Then materials were processed in a 20 mm corotating twin-screw extruder with a length-to-diameter (L/D) ratio of 40:1. The barrel temperatures of the extruder were controlled at 170–210 °C. The extruded strand passed through a water bath and was subsequently pelletized. The pellets were dried for about 4 h before being injection molded. The temperature used for injection-molded specimens was 190–230 °C from feed zone to die zone, and the injection pressure was 95 bar, the holding pressure was 50 bar, and the cooling time was 10 s.

### 2.3. Mechanical Testing

The tensile tests were conducted on the universal testing machine (AGS-X, Shimadzu) with the GB/T 1040-2018. The specimens of dimension 75 mm × 10 mm × 4 mm were used for analysis. The sample of 75 mm length was clamped into the 2 jaws of the machine. Each end of the jaws covered 20 mm of the sample. Tensile strength specimens were tested with a tensile speed of 50 mm/min, and tensile modulus specimens were tested with a tensile speed of 1 mm/min. The three-point bend flexural test was conducted following GB/T 9341-2008 using the universal testing machine (AGS-X, Shimadzu, Japan) at a rate of 2 mm/min. The specimens of dimension 80 mm × 10 mm × 4 mm were used for analysis. The Izod notched impact resistance was performed according to GB/T 1843-2008 by an impact tester (ZBC 50, Nss (Shenzhen) Laboratory Equipment Co., LTD, China). The specimens of dimension 80 mm × 10 mm × 4 mm and the notch bottom radio is 0.25 mm were used for analysis. Five samples were tested for each type of composite material, and the average values were reported.

### 2.4. Morphology Analysis

The morphology of the impact specimens’ surfaces was studied utilizing scanning electron microscopy (SEM, Phenom XL) under an acceleration voltage of 15 kV. The test specimens were sputtered with gold to eliminate the electron charging effects. The objective was to acquire information regarding the materials’ filler dispersion and bonding quality.

### 2.5. Thermal Properties

#### 2.5.1. Differential Scanning Calorimetry (DSC)

The composites were analyzed by differential scanning calorimetry (DSC) with a thermal analyzer (DSC204F1 Phoenix). The measurements were under the protective atmosphere of nitrogen, take 5–10 mg of additives to heat from room temperature to 200 °C quickly. Keep 200 °C for 3 min to eliminate the thermal history of the sample. Then cool down from 200 °C to 20 °C with a 10 °C /min cooling rate, and finally heating from 20 °C to 200 °C at 10 °C /min to determine the non-isothermal crystallization and melting behavior of the material.

The degree of crystallinity (*X_c_*%) was determined from the second melting enthalpy values using the following equation:

(1) $$\;\chi_{c}=\frac{\Delta H_{m}}{\Delta H_{m}^{o}}\times \frac{1}{W_{f}}\times 100\left(\%\right)$$

where $\Delta H_{m}$ is melting enthalpy of the specimens (J/g), $\Delta H_{m}^{o}$ is the enthalpy value of melting of a 100% crystalline form of PP (209 J/g), and $W_{f}$ is the weight fraction of polymer into the composite material.

#### 2.5.2. Thermogravimetric Analysis (TGA)

The composites were analyzed by thermogravimetric analysis (TGA) with a thermal analyzer (TGA/DSC 3+, Mettler Toledo). Under the argon’s protective atmosphere, 5–10 mg of materials were added to the alumina crucible, and the temperature was increased from 40 °C to 600 °C to keep the heating rate of 10 °C/min to study the thermal decomposition performance.

### 2.6. Water Absorption

Materials were cut into small pieces of 20 × 20 × 1 mm^3^, soaked in water for 30 days, and maintained the temperature at 25 °C. Their weight gain was periodically measured. The water absorption is calculated according to the following equation:

(2) $$\;WA=\frac{M_{e}-M_{0}}{M_{0}}\times 100\left(\%\right)$$

where $M_{e}$ is the mass of the sample after water absorption, $M_{0}$ is the original mass of the sample.

### 2.7. Antibacterial Assay

The antibacterial activity of the sample was determined by the mycelial growth rate method. Taking the free growth of bacteria as a blank pair, the sample size is 36 mm^2^ × 1 mm, the distance between the sample and the center of the bacteria is 3.5 cm, and the measurement is repeated three times for each sample. Seal the culture dish planted with bacteria with a parafilm and place it upside down in a 28 °C constant temperature incubator. When the control colony grows to 2/3 of the diameter of the culture dish, use the cross method to measure the colony diameter and calculate it according to the following formula Inhibition rate.

(3) $$r_{I}=\frac{\left(D_{o}-d\right)-\left(D_{s}-d\right)}{\left(D_{o}-d\right)}\times 100\%$$

$r_{I}$ is the growth inhibition rate, $D_{o}$ is the control colony diameter, d is the bacterial cake diameter, $D_{s}$ is colony diameter with the sample.

## 3. Results and Discussion

### 3.1. Morphology

**Figure 1:**
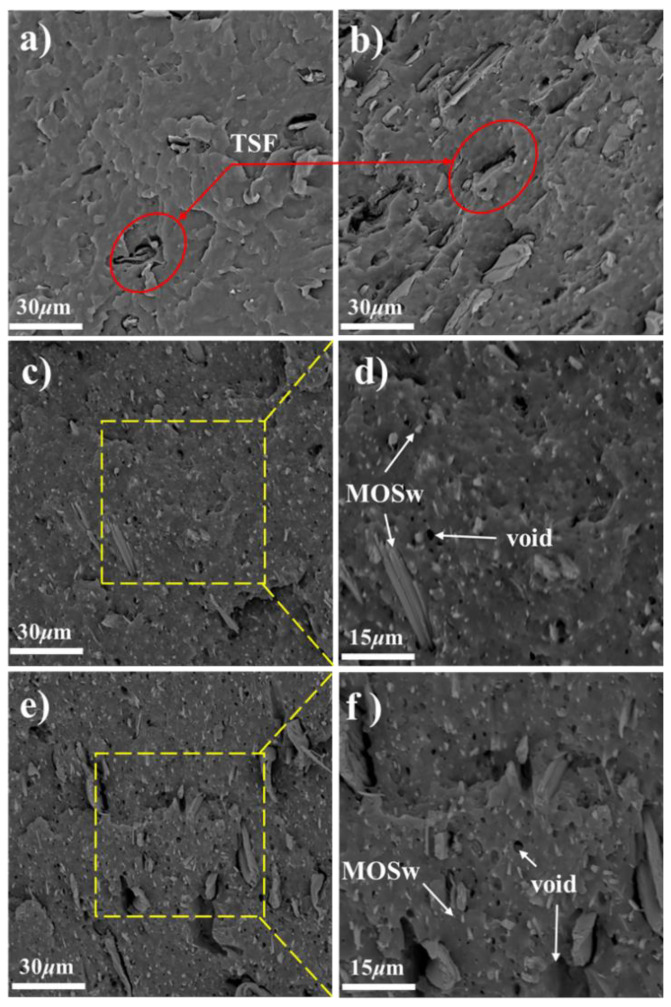
SEM images of samples of B (**a**), C (**b**), D (**c**,**d**), E (**e**,**f**).

The property of the composites is related to the interface compatibility and adhesion between the filler and the matrix. SEM characterization is an effective means to observe the morphology and interface of the material. Impact section morphology of TSF/rPP and TSF/MOSw/rPP composites are shown in Figure 1. The filler was arranged in parallel with the flow direction of the matrix and was evenly dispersed in the matrix. Figure 1a,b are SEM images of composite B and C, respectively. The TSF had uniform dispersion in the rPP matrix. The brittle cross-section was flat, and no voids formed from the TSF pulled out, the composite showing a typical brittle fracture section. It indicated a good relationship between TSF and matrix interface compatibility. Figure 1c is an SEM image of composite D. There are apparent voids in the section surface because of the whisker’s pullout, caused by poor interface adhesion between MOSw and rPP matrix.

Figure 1b,e are microscopic morphology of composite C and E filled with 30 wt% filler, respectively. At the same content of filler, the fracture surface of E could be observed the gap between the TSF and the rPP matrix and the voids formed by TSF pullout, compared to composite C. It states that after the addition of MOSw, the interface compatibility among the components became worse.

### 3.2. Thermal Properties

#### 3.2.1. Differential Scanning Calorimetry (DSC)

**Figure 2:**
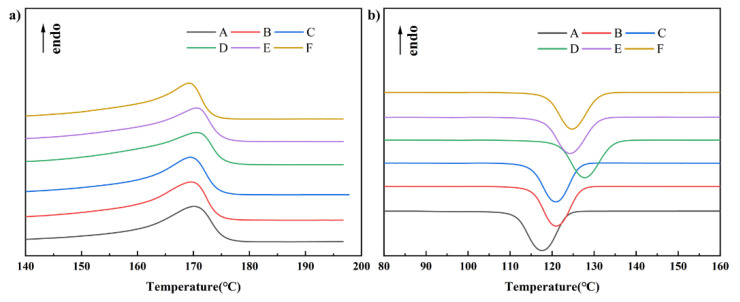
(**a**) composites’ second melting peak temperatures. (**b**) the crystallization peak temperatures of composites.

The sample was characterized by differential scanning calorimetry (DSC) to confirm the influence of TSF and MOSw to rPP on the composites’ crystallization ability and melting behavior. The results of the DSC analysis are given in Figure 2 and Table 2, where Tm is melting temperature, ΔHm is melting enthalpy, Tc is the temperature of crystallinity and Xc is a degree of crystallinity.

The melting temperature of composites was around 169–170 °C and did not change significantly with the increase of TSF content. Meanwhile, the addition of TSF significantly changed the crystallization process of composites. First, the crystallization temperature of the composites has increased from 117.7 °C for rPP material to 120.6 °C for 30 wt% TSF/rPP composite material. Then, the material exhibited higher crystallinity with the higher TSF content. These proved that TSF could be used as a nucleating agent of rPP, to promote heterogeneous nucleation and increase the crystallization temperature and crystallinity of the material.

Single-filled MOSw can promote the crystallization of PP. The addition of 20 wt% MOSw makes the crystallization temperature of the composite G reach 127.6 °C, and the crystallinity reaches 38.91%. However, the crystallization temperature and crystallinity both decreased after adding TSF. This phenomenon could be due to the co-filling of TSF and MOSw, which makes the interface compatibility of the material worse, and more defects are generated in the matrix and hinder the crystallization of rPP.

#### 3.2.2. Thermogravimetric Analysis (TGA)

**Figure 3:**
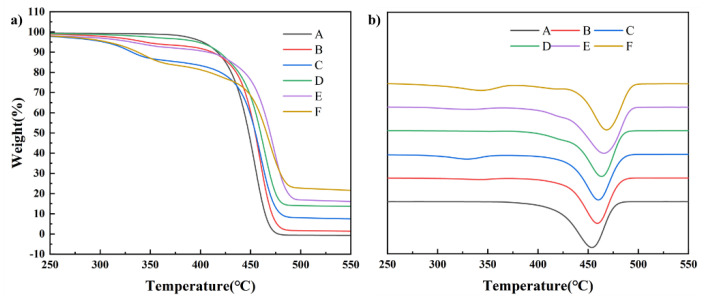
The (**a**) TGA and (**b**) DTA curves of composites.

Figure 3 shows the thermogravimetric analysis (TGA) and differential thermal analysis (DTA) curves of composites. The addition of TSF caused the degradation of TSF/rPP composites to be divided into two stages. In the composites B and C, the thermal degradation in the first stage between 258 °C and 379 °C is mainly attributed to TSF degradation, the degradation of rPP caused the second stage of pyrolysis between 379 °C and 495 °C. However, in composites D to F, the first stage was also caused by TSF, while the second stage, between 384–432 °C, was caused by MOSw degradation, and the third stage was rPP degradation.

The TGA data of composites are shown in Table 3. The T_5_, T_50_, and T_75_ represent the temperature at which composite mass degrades 5%, 50%, and 75%, respectively. When the addition of TSF and MOSw would reduce the initial decomposition temperature of the composites. However, relative to the maximum weight loss rate point temperature ($T_{d}^{peak}$) of 454.78 °C of rPP, the $T_{d}^{peak}$ of composite E has reached 473.55 °C. The decrease in the initial degradation temperature of the material could be due to TSF and MOSw degradation temperature being inferior to the matrix. Furthermore, the gap between the filler and the matrix promotes heat conduction in the matrix. Especially when TSF and MOSw were co-filled, the interface compatibility of the materials became worse, the initial degradation temperature of the composite decreased significantly. On the other hand, the degradation of MOSw would absorb heat, and the carbon produced by the degradation of TSF will hinder the further degradation of the matrix, thereby increasing the $T_{d}^{peak}$ of the composites.

### 3.3. Mechanical Properties

#### 3.3.1. Tensile Property

**Figure 4:**
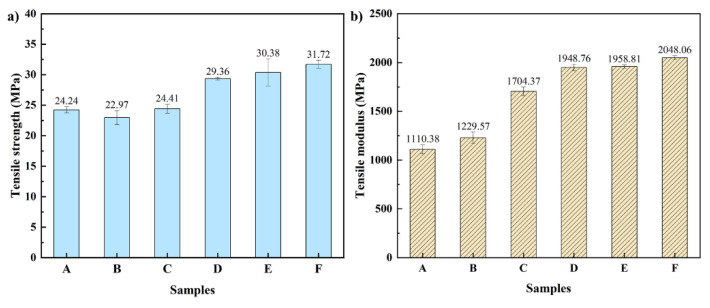
(**a**) Tensile strength and (**b**) tensile modulus of the composites.

The results of the tensile test are shown in Figure 4. When filled 30 wt% TSF, the tensile strength of the composites reached 24.41 MPa, which is similar to the 24.24 MPa of rPP. It reveals that the addition of TSF did not significantly change the tensile strength of rPP. Tensile modulus was shown in Figure 4b. The TSF could increase of tensile modulus of rPP. When filled 30 wt% TSF, the tensile modulus of the composites reached 1704.37 MPa, an increase of 53.49% compared to composite A. The significant increase in tensile modulus is mainly connected with the rigidity of TSF.

Adding MOSw to polypropylene can enhance the mechanical properties of the composites, such as tensile strength, flexural strength, and flexural modulus. The composite D with 20 wt% MOSw added, when the external force is applied, the external load is transferred to the MOSw through the matrix so that the whiskers were pullout along with the direction of the force. Which could consume energy, slow down the crack growth, and improve the material’s tensile properties. Thus, the tensile strength of F relative to C is increased by 30.86%, reaching 31.72 MPa; furthermore, the tensile modulus also reaches 2048.06 MPa. This great reinforcement is mainly attributed to the MOSw’s high mechanical strength and modulus and aspect ratios.

#### 3.3.2. Flexural Property

**Figure 5:**
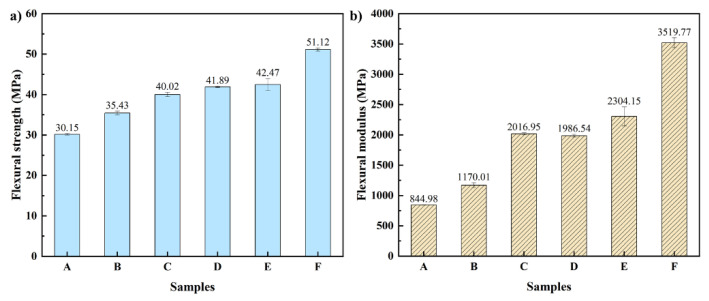
(**a**) Flexural strength and (**b**) flexural modulus of the composites.

The flexural strength and flexural modulus of composites with different loading levels of TSF and MOSw are shown in Figure 5. The flexural strength of the composites increases with the increase of the TSF content. Compared to rPP, the flexural strength of the composites material with 30 wt% TSF reached 40.02 MPa, improved 32.74%. Compared with rPP, the flexural strength of the composite with 20 wt% MOSw increased from 30.15 MPa to 41.89 MPa. The flexural modulus of TSF/rPP and TSF/MOSw/rPP series composites presented an upward trend. Compared with rPP, the flexural modulus of composite F and M increased 138.69% and 316.55%, respectively, reaching 2016.95 MPa and 3519.77 MPa, while the flexural modulus of rPP was only 844.98 MPa.

This phenomenon can be attributed to TSF and MOSw were arranged in parallel in the matrix along the flow direction during injection molding. The external load was transmitted to the filler through the matrix when the sample was subjected to the flexural test. The energy was consumed by pullout and fracture of TSF and MOSw, thereby improving the flexural strength flexural modulus of the composites. Moreover, TSF and MOSw re-strict the mobility of the macromolecules of the PP matrix when subjected to tensile stress, which also increases the bending properties of the composites.

#### 3.3.3. Impact Strength

**Figure 6:**
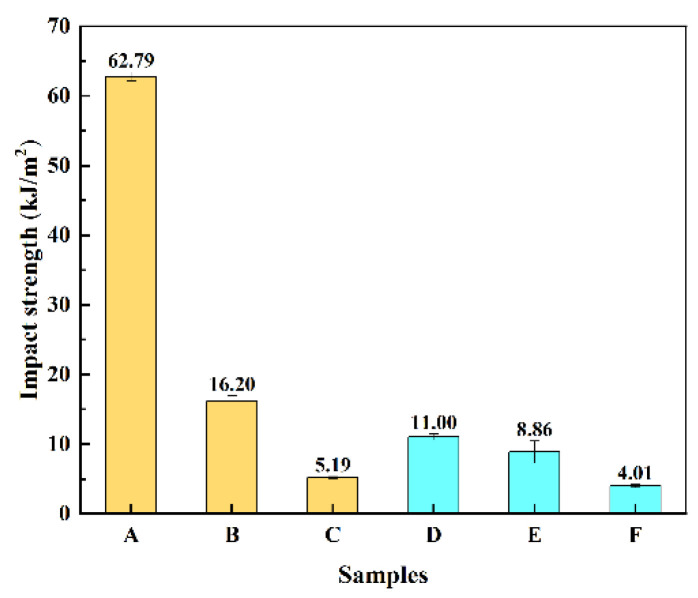
Impact strength of the composites.

The results of the cantilever beam notched impact strength test of TSF /rPP and TSF/MOSw/rPP are shown in Figure 6. The impact strength of TSF/rPP composite decreased rapidly when TSF adds. After 10 wt% TSF filled, the impact strength reduces from 62.60 KJ/m^2^ of A to 16.31 KJ/m^2^ with composite B. The impact strength of composite D is 10.76 KJ/m^2^ decreased by 82.81% compared to composite A.

This result depends on factors such as fiber to matrix compatibility, matrix defects, and fiber crystalline morphology.TSF and MOSw were stiffness materials. The introduction of TSF and MOSw into the rPP matrix would increase the rigidity and brittleness of the materials and reduce the material’s tenacity. The defects caused by filler and filler aggregation also be a dedication factor in the reduced impact strength of the composites.

### 3.4. Water Absorption

**Figure 7:**
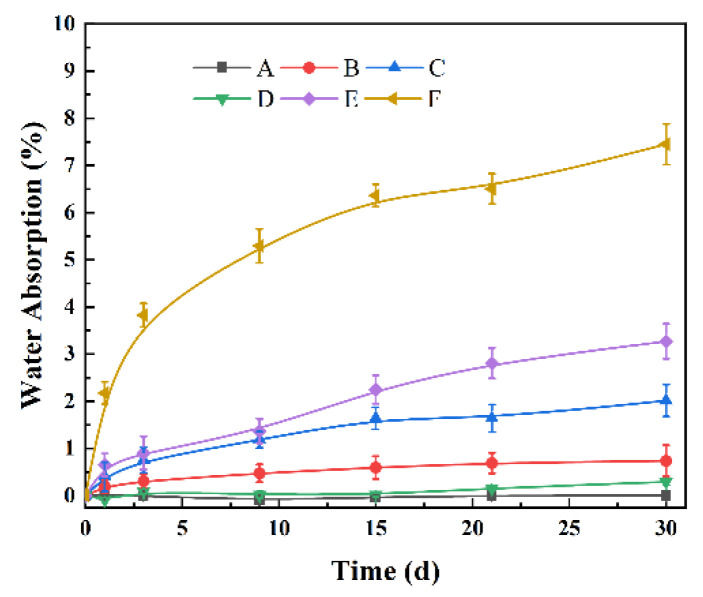
Water absorption of the composites.

The curve of water absorption of TSF/rPP composites with time is shown in Figure 7. The rPP that composite A does not absorb water even when immersed in water. The water absorption rates of composites B and C with 10 wt% and 20 wt% TSF added are 0.73% (30 d) and 2.02% (30 d), respectively. It states that with the increase of TSF content, the water absorption of composites also gradually increases. This phenomenon can be attributed to the hydrophilic nature of TSF, which leads to absorbing water from the environmental condition. The hydrophilic nature of TSF is from the hydroxyl groups in the cellulose structure, which attract water molecules and bind with them through hydrogen bonding.

The 20 wt% MOSw filled to rPP did not extraordinarily promote the composite’s water absorption rates increase, which was around 0.29%. However, with the same content TSF, the water absorption of reinforced hybrid composites is higher than composites with single TSF fill. For example, the water absorption of composite F reached 7.45% (30 d), which is 268.81% higher than that of composite C. The higher water absorption may be caused by poor compatibility of the filler and the matrix interface in the composite after being filled with TSF and MOSw. In addition, the terrible interface compatibility increased gaps between the fillers and matrix, caused water to be more accessible infiltrate to the composite, and increased water absorption rate.

### 3.5. Antibacterial Assay

It has been reported that the combination of recycled plastic and degradable natural plant fiber can be used as a container for plant nurseries, and inhibiting bacterial infection has an important impact on the cultivation of crops. At the same time, the bacteria’s erosion of tobacco stalk flour components in products will also accelerate the deterioration of material properties. Colletotrichum micotianae Averna was selected here, and the inhibitory effect of the tobacco stalk composite material on the growth of bacteria was observed.

**Figure 8:**
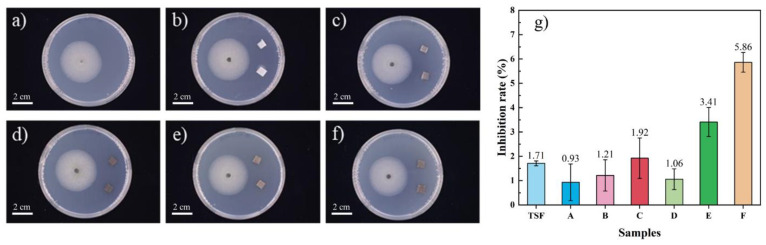
The influence of different samples on the colony growth of Colletotrichum micotianae Averna, (**a**) Control group; (**b**) sample A; (**c**) sample B; (**d**) sample C; (**e**) sample E; (**f**) sample F, and the (**g**) Inhibition rate of different samples to Colletotrichum micotianae Averna.

Figure 8 shows TSF has an antibacterial effect on Colletotrichum micotianae Averna, with an antibacterial rate of 1.71%. After being added TSF to rPP, the inhibitory effect still exists. Composite C, which added 30 wt% TSF, the antibacterial rate was 1.92%. Meanwhile, the antibacterial effect of the composite material filled with TSF and MOSW is better than that of a single filler filling. For example, compared with composite C, the antibacterial rate of composite E reached 5.86% after adding 20 wt% MOSw. This result may be that after the material absorbed water, the antibacterial substances in the TSF are dissolved into the culture medium, inhibiting the growth of Colletotrichum micotianae Averna. The antibacterial components in the composite mainly come from bioactive substances such as alkaloids, polyphenols, and flavonoids in the tobacco stalk. Therefore, the degree of separation out of these biologically active substances will affect the antibacterial effect of the material. Due to the addition of MOSW, F composite material has higher water absorption than C, cause active substances are more straightforward separate out of composite, making the antibacterial effect of F better than C composite, which was only filled with tobacco stalk powder.

## 4. Conclusions

The study has shown that recycled polypropylene-based hybrid composites consisting of TSF and MOSw fiber can be fabricated and achieve a balance of properties such as high stiffness, low price, and antibacterial ability. The following conclusions could be drawn from the results of the present study:

The tobacco stalk flour has good adhesion with recycled polypropylene. The crystallinity of recycled PP properties was increased by adding TSF, proving tobacco stalk flour could be used as a nucleating agent of PP. Moreover, TSF and MOSw would reduce the initial degradation temperature but increase the $T_{d}^{peak}$ of the composites from the TGA results.

The flexural modulus and flexural strength of composites would improve when reinforced with TSF and MOSw. The stiffness of the TSF/PP composite increased with the addition of TSF. This was possibly from poor interphase adhesion as observed in SEM micrographs and strength concentration caused by fiber aggregation, thus resulting in the reduced toughness of the composite.

The water absorption at saturation increased markedly with the increase of TSF. The antibacterial assay shows that tobacco stalk flour could bring antibacterial properties to composite materials.

Hence, adding tobacco stalk flour to rPP could recycle waste, reduce environmental pollution, and obtain composite material with excellent mechanical properties and antibacterial ability provided a new development idea for tobacco stalks recycling and applications.

## Figures and Tables

**Table 1:** Formulations of the composites.

	A	B	C	D	E	F
rPP (wt%)	100	88	68	78	68	48
MOSw (wt%)	0	0	0	20	20	20
TSF (wt%)	0	10	30	0	10	30
MAPP (wt%)	0	2	2	2	2	2

**Table 2:** Thermal parameters of the investigated composites.

	Composite	TSF Content (wt%)	Tm (°C)	ΔHm (J/g)	Tc (°C)	ΔHc (J/g)	Xc (%)
rPP/TSF	A	0	170.6	68.72	117.7	−79.49	32.88
	B	10	169.6	62.69	120.6	−67.71	33.32
	C	30	169.6	52.37	120.6	−56.33	35.79
	D	0	170.6	65.06	127.6	−66.25	38.91
	E	10	170.6	48.80	124.4	−51.02	33.35
	F	30	169.3	36.49	124.7	−37.81	34.91

**Table 3:** The TGA data of composites.

	Composite	TSF Content (wt%)	T_5_ (°C)	T_50_ (°C)	T_75_ (°C)	$T_{d}^{peak}({}^{\circ}C)$
rPP/TSF	A	0	402.58	448.35	457.65	454.78
	B	10	344.92	454.66	463.65	459.56
	C	30	303.85	454.98	466.32	460.36
rPP/MOSw/TSF	D	0	397.65	460.15	471.41	463.59
	E	10	331.21	469.63	481.95	473.55
	F	30	307.21	466.03	485.57	468.22

## Data Availability

The data presented in this study are available on request from the corresponding author.
